# Epigenetic Control of Gene Expression in the Alcoholic Brain

**DOI:** 10.35946/arcr.v35.1.08

**Published:** 2013

**Authors:** Igor Ponomarev

**Affiliations:** **Igor Ponomarev, Ph.D.,***is a research assistant professor at the Waggoner Center for Alcohol and Addiction Research and the College of Pharmacy, University of Texas at Austin, Austin, Texas.*

**Keywords:** Alcohol consumption, alcoholism, chronic alcohol exposure, alcohol use, abuse and dependence, epigenetics, epigenetic therapeutics, gene expression, brain, brain cells, brain pathology, behavior, DNA methylation, histone, microRNA, transcription, pharmacotherapy, animal models, human studies

## Abstract

Chronic alcohol exposure causes widespread changes in brain gene expression in humans and animal models. Many of these contribute to cellular adaptations that ultimately lead to behavioral tolerance and alcohol dependence. There is an emerging appreciation for the role of epigenetic processes in alcohol-induced changes in brain gene expression and behavior. For example, chronic alcohol exposure produces changes in DNA and histone methylation, histone acetylation, and microRNA expression that affect expression of multiple genes in various types of brain cells (i.e., neurons and glia) and contribute to brain pathology and brain plasticity associated with alcohol abuse and dependence. Drugs targeting the epigenetic “master regulators” are emerging as potential therapeutics for neurodegenerative disorders and drug addiction.

Whether a specific gene is transcribed or repressed is determined by the specific status (i.e., conformational state) of the complex of chromosomal DNA and proteins (i.e., the chromatin) and by the recruitment of specific proteins (i.e., transcription factors) to regulatory sites on the DNA (Copeland et al. 2010). Chromatin states can change as a result of enzyme-mediated covalent modifications of the DNA and structural chromatin proteins (i.e., histones) (Borrelli et al. 2008; Copeland et al. 2010). These changes in chromatin, which are often termed epigenetic marks, include such modifications as DNA methylation and histone methylation and acetylation. In addition, incorporation of histone variants, adenosine triphosphate (ATP)-dependent chromatin remodeling, and regulation of gene expression by noncoding RNAs also are considered epigenetic phenomena and play important roles in regulation of gene expression. It is becoming increasingly clear that epigenetic mechanisms play a key role in cellular differentiation and regulation of cell type–specific transcriptional programs, producing a remarkable heterogeneity of cellular transcriptomes^1^ that reflect the physiological properties and functional state of individual cells.

The brain arguably is one of the most complex biological tissues and enables the organism to sense, remember, and respond to its environment. It constantly adapts to environmental stimuli through regulated changes in gene expression. Chronic alcohol exposure causes wide-spread changes in brain gene expression in humans and animal models (Mulligan et al. 2006; Ponomarev et al. 2012), and there is evidence that many of these changes mediate the processes of cellular adaptation leading to addiction (Mayfield et al. 2008). Until recently, the role of epigenetic processes in alcohol’s effects on the central nervous system (CNS) has been largely understudied. However, in the past 5 years the number of studies that suggested a role for epigenetics in alcohol-related molecular and behavioral changes has grown considerably. This review summarizes evidence for the role of epigenetic modifications in alcohol’s effects on brain gene expression and behavior.

## DNA Methylation

DNA methylation generally is associated with transcriptional repression. It mainly occurs at sites where a cytosine and a guanosine nucleotide are located next to each other (i.e., CpG dinucleotides). If these CpG dinucleotides are located within regulatory sequences, such as promoter regions, their methylation can block the binding of transcription factors and/or establish a repressive chromatin state (Renthal and Nestler 2009*b*). One of the first indications that DNA methylation may play a role in alcoholism can be traced back to 1940s and 1950s, to the work of Dr. Roger J. Williams, a biochemistry professor at the University of Texas at Austin. He showed for the first time that dietary changes could affect beverage alcohol (i.e., ethanol) consumption in rodents. Specifically, diets deficient in B vitamins (e.g., folic acid and choline) increased consumption of solutions containing 10 percent ethanol in some rats, whereas vitamin-enriched diets decreased it (Williams et al. 1949). It now is well established that folates and several other B vitamins are critical for one-carbon metabolism and the synthesis of a compound called *S*-adenosyl-methionine (SAM), which serves as the primary methyl group donor in most transmethylation reactions, including DNA methylation (Hamid et al. 2009). Therefore, it is possible that dietary changes in this early study affected alcohol consumption via changes in DNA methylation and methylation-regulated gene expression.

Chronic alcohol consumption causes well-documented vitamin B and folate deficiencies that negatively affect the biochemical reactions in which a chemical unit containing one carbon atom (e.g., a methyl group) is transferred through several steps from a donor to another compound, such as DNA (i.e., one-carbon metabolism). These effects on one-carbon metabolism can result in excess levels of the SAM precursor homocysteine in the blood (i.e., homo-cysteinemia) and decreased SAM production (Blasco et al. 2005; Hamid et al. 2009). In addition, alcohol can affect DNA methylation through several other mechanisms, including the following:

- The alcohol metabolite, acetalde-hyde, may induce inhibition of an enzyme called DNA methyltransferase 1 (DNMT1) that mediates most DNA methylation reactions needed to maintain the cell’s normal functioning (Garro et al. 1991).
- Alcohol-induced DNA damage and the resulting repair reactions can lead to demethylation of 5-methylcytosine nucleotides (Chen et al. 2011).

Both of these mechanisms can cause reduced levels of methylation throughout the DNA (i.e., global DNA hypomethylation), a chromatin state associated with many pathological conditions, including cancer (Pogribny and Rusyn 2012). Alcohol-induced global DNA hypomethylation has been reported in several peripheral tissues of alcohol-related models and may play a role in alcoholic liver disease, fetal alcohol syndrome, and colon cancer (Choi et al. 1999; Garro et al. 1991; Hamid et al. 2009; Lu et al. 2000; Shukla et al. 2008). However, the effect of chronic alcohol on global DNA methylation seems to be tissue specific because one study reported enhanced DNA methylation (i.e., global DNA hypermethylation) in a certain type of blood cells (i.e., peripheral mononuclear cells) in alcoholic patients undergoing early alcohol withdrawal (Bonsch et al. 2004).

Two recent studies (Manzardo et al. 2012; Ponomarev et al. 2012) have examined alcohol’s effects on global DNA methylation in the brain. Both studies measured DNA methylation in the frontal cortex of chronic alcoholics and matched control cases, but using two different methods. Ponomarev and colleagues (2012) studied genomic regions that included DNA sequences called long terminal repeat (LTR)-containing retrotransposons, also known as endogenous retroviruses (ERVs), most of which are nonfunctional remnants of ancient retroviral infections (Antony et al. 2004). The investigators showed that these repeats, which usually are heavily methylated, were less methylated in alcoholic brains, which was associated with their increased expression. Because ERVs constitute a significant part of the human genome, the study concluded that alcohol abuse causes global DNA hypomethylation in the brain, which is consistent with the majority of previous studies on alcohol-induced changes in DNA methylation. Manzardo and colleagues (2012) used immunological methods (i.e., immunoprecipitation) to isolate methylated DNA from alcoholics and control subjects and then applied this DNA to microarrays containing genomic promoter regions to identify promoters for which the methylation patterns differed between the two groups. The analyses found no differences between the groups in total methylation at the whole-genome level; however, about 20 percent of all promoters were differentially methylated between the groups, with less than half of these promoters showing greater methylation in alcoholics.

These complementary findings suggest that chronic alcohol causes a general decrease in the overall number of methylated cytosines but also could lead to the de novo methylation of previously unmethylated nucleotides at the promoters of some genes. Such a combination of these processes already has been widely reported in studies of cancer, showing, for example, that methyl-deficient diets induce development of liver tumors (i.e., hepatocarcinogenesis) associated with global DNA hypomethylation and promoter hypermethylation at specific genes (Ehrlich 2005; Pogribny and Rusyn 2012). Hypomethylated states associated with cancer and other pathological conditions often are accompanied by a downregulation of the gene encoding DNMT1 (Hervouet et al. 2010), which also has been observed in the brains of chronic alcoholics (Ponomarev et al. 2012). These striking similarities point to some common mechanisms of methyl deficiency across tissues.

Studies assessing epigenetic regulation of individual genes in the brain have shown that alcohol’s effects on DNA methylation depend on a variety of factors, including the specific gene targets, developmental stage of exposure, and type of neuronal tissue affected. Much of this work has focused on the central effects of prenatal alcohol exposure and on gene regulation in cell cultures. Prenatal exposure of rats to alcohol resulted in DNA hypermethylation and a reduced expression of a protein called brain-derived neurotrophic factor (BDNF) in olfactory bulbs of rat pups, which was associated with loss of neurons in this brain region (Maier et al. 1999). Similar molecular results were obtained in a separate study where prenatal alcohol treatment of rats led to DNA hypermethylation and a decreased expression of a protein characteristically found in brain cells called astrocytes (i.e., glial fibrillary acidic protein [GFAP]) in the brains of the pups (Valles et al. 1997). In neural cell cultures, alcohol-induced downregulation of cell-cycle genes was paralleled by an increased DNMT activity and hypermethylation of the promoters of those genes (Hicks et al. 2010). Conversely, upregulation of the gene encoding a receptor subunit for the neurotransmitter glutamate (i.e., the NMDA NR2B receptor subunit) was associated with demethylation of CpG dinucleotides in the gene’s promoter after chronic alcohol (Marutha Ravindran and Ticku 2004).

However, some reports suggest that the relationship between DNA methylation and the expression of neighboring genes may be even more complex than previously thought (Ehrlich 2005). For example, a recent study demonstrated an increased expression of a signaling molecule called prodynorphin (PDYN) that was associated with methylation of a CpG dinucleotide located in a DNA region behind the actual protein-coding region of the gene (i.e., in the 3′-untranslated region of the gene) in the brains of alcohol-dependent people (Taqi et al. 2011), although no causal link was established.

Specific DNA methylation patterns differ among tissues and cell types, and these differences contribute to establishing the cells’ epigenetic landscape and transcriptional programs and defining cellular identity (Bernstein et al. 2007). Also, although alcohol’s general effects on DNA methylation may be similar across various tissues, the specific genes affected by this regulation may differ depending on cell type. The epigenetic regulation of such proteins as GFAP, which is a marker of astrocytes, and the NR2B subunit, which generally is expressed in neurons, suggests that alcohol-induced epigenetic changes will affect molecular markers of individual cell types to a greater degree than other proteins. Many studies of alcohol’s epigenetic modification of the chromatin have been conducted in blood cells obtained from alcoholics (Biermann et al. 2009; Bonsch et al. 2005; Hillemacher et al. 2009). Because of the concern regarding the tissue specificity of alcohol’s epigenetic effects, however, the results of these important studies cannot be readily generalized to mechanisms in brain. Therefore, parallel measurements of the entirety of all alcohol-induced epigenetic changes (i.e., the epigenomic changes) in the blood and brain should be obtained and vigorously compared in animal models to detect common patterns, based on which generalization of results in humans can be made.

## Histone Modifications

Histone proteins are the second major target of epigenetic changes. These proteins can be modified by a relatively large number of specific enzymes that mediate covalent attachment and removal of four classes of chemical groups: methyl, acetyl, phosphate, and ubiquitin (Bernstein et al. 2007; Borrelli et al. 2008). Studies of alcohol-induced modifications mainly have focused on two histone modifications: a trimethylation of histone 3 at the lysine 4 residue (H3K4me3), which is a promoter-enriched chromatin mark of actively transcribed genes, and acetylation of various residues of histones 3 and 4 (H3 and H4). Histone acetylation generally is associated with a more open, accessible structure of the chromatin and, consequently, increased transcription, whereas deacetylated histones can cause transcriptional repression (Bernstein et al. 2007).

Chronic alcohol abuse in humans can result in global and gene-specific increases in H3K4me3 in the brain cortex (Ponomarev et al. 2012) and in either increases or decreases of this modification in promoters of specific genes in the hippocampus (Zhou et al. 2011). The latter study used a combination of two techniques (i.e., chromatin immunoprecipitation followed by DNA sequencing [ChIP-Seq]) to detect individual genes with differences between alcoholics and control subjects in H3K4 promoter trimethylation and in parallel measured the levels of transcription of the same genes. Interestingly, differences in promoter methylation did not correlate with differences in gene expression, suggesting that H3K4me3 status alone is not a reliable predictor of genome-wide steady-state mRNA levels at a given time point. A possible explanation of these results is that the H3K4me3 mark in the promoter regions only indicates that the chromatin is in an open conformation that is accessible to regulatory or transcription factors but does not mean that transcription actually is initiated and the transcription machinery is present (Bernstein et al. 2007). A recent study (D’Addario et al. 2011) supports this hypothesis as well as previous findings showing mechanistically linked but temporally complex relationships between chromatin marks at gene promoters and mRNA abundance. The investigators explored the effects of ethanol and its metabolite acetalde-hyde on various chromatin marks and the transcription of the *PDYN* gene in a human cell line derived from a tumor arising from nerve tissue cells (i.e., a neuroblastoma). The analyses suggested that the ethanol-induced increase in H3K4me3 that was observed after 72 hours of ethanol exposure did not result in initiation of *PDYN* transcription but kept the gene in a poised state for later reactivation. This is consistent with other findings regarding *PDYN* activation in human alcoholics (Taqi et al. 2011).

Most evidence to date on the role of central epigenetic processes in alcoholism has been collected from studies focusing on histone acetylation, often by modifying the activities of the enzymes that add acetyl groups (i.e., histone acetyl transferases [HATs]) or remove acetyl groups (i.e., histone deacetylases [HDACs]). Particularly, small molecules that inhibit HDAC function (HDACis) and thus result in increased histone acetylation have been investigated intensely in recent years. These molecules are attractive because they can enter the brain via the blood (i.e., cross the blood–brain barrier) and exert a broad range of effects in the CNS, including enhanced memory formation as well as anti-inflammatory and neuroprotective effects (Kazantsev and Thompson 2008; Sweatt 2009). Several studies using HDACis demonstrated effects of altered histone acetylation on different alcohol-related behaviors, including withdrawal-related anxiety (Pandey et al. 2008), locomotor sensitization (Sanchis-Segura et al. 2009), alcohol consumption (Wostenholme et al. 2011), conditioned place aversion (Pascual et al. 2012), and rapid tolerance (Sakharkar et al. 2012). For example, Pandey and colleagues (2008) showed that acute ethanol increased H3K9 and H4K8 acetylation in rats, whereas anxiety-like behaviors during withdrawal after chronic alcohol exposure were associated with decreases in these acetylation marks, decreased expression of several proteins (e.g., CREB-binding protein [CBP] and neuropeptide Y [NPY]), and increased HDAC activity. However, treatment with the HDACi, trichostatin A (TSA), to block HDAC activation prevented the deficits in gene expression and the development of withdrawal-related anxiety. Sanchis-Segura and colleagues (2009) demonstrated that treatment of mice with another HDACi (i.e., sodium butyrate) altered some alcohol-related behaviors (e.g., enhanced ethanol-induced locomotor sensitization) but had no effect on others (e.g., ethanol tolerance or withdrawal). Finally, daily injections of TSA in mice that had continuous access to both water and an alcohol solution increased the animals’ alcohol consumption (Wolstenholme et al. 2011).

Similar to DNA methylation, alcohol’s effects on histone acetylation are tissue, brain region–, and cell type–specific. For example, a single dose of ethanol^2^ into the stomach increased the levels of H3 acetylation in the liver, lungs, and testes but had no effects in other tissues, including whole brain, of rats (Kim and Shukla 2006). In the brain, ethanol-induced changes in H3/H4 acetylation were observed in the central and medial but not the basolateral nuclei of the amygdala (Pandey et al. 2008; Sakharkar et al. 2012); moreover, the increased histone acetylation appeared to be specific for neurons (Sakharkar et al. 2012).

Other factors that can affect alcohol-induced changes in histone acetylation include species, the organism’s specific genetic makeup (i.e., genotype), age, the dose and route of ethanol administration, and duration of exposure. For example, ddY mice treated with chronic ethanol vapor showed increases of both global and gene-specific histone acetylation in the ventral midbrain during withdrawal that peaked around 10 hours post ethanol (Shibasaki et al. 2011). Also, intermittent alcohol exposure produced different effects on his-tone acetylation in adolescent and adult rats, with juvenile animals generally showing more changes (Pascual et al. 2009, 2012). Consistent with these studies was the finding that ethanol exposure during the early postnatal period in rats resulted in a marked reduction of CBP levels and histone acetylation in the developing cerebellum (Guo et al. 2011). In addition, possible interactions among various factors may result in different time courses for alcohol-induced changes, because histone acetylation measured 24 hours after the last of repeated alcohol injections was increased in some brain areas (e.g., frontal cortex and nucleus accumbens), decreased in others (e.g., striatum), and unchanged in still others (e.g., hippocampus) (Pascual et al. 2009).

Histone acetylation generally is associated with transcriptional activation, but similar to the H3K4me3 mark, the relationships between levels of histone acetylation and steady-state mRNA are complex, because activation of different genes is associated with acetylation of different residues of H3 and H4 at different time points (Renthal and Nestler 2009*a*). And although alcohol’s effects on histone acetylation now are well established, the exact mechanisms underlying this influence on gene expression are not well understood. Alcohol-induced changes in histone acetylation are paralleled by regulation of several genes, including *CBP, NPY* (Pandey et al. 2008), *FosB* (Pascual et al. 2012), and *NR2B* (Qiang et al. 2011). One proposed mechanism involves the transcription factor CREB, to which CBP can bind (Moonat et al. 2010). CBP has intrinsic HAT activity and, when recruited by CREB, can promote transcriptional activation by acetylating histones. This mechanism has been shown to play a role in cocaine-induced regulation of *FosB* (Levine et al. 2005). A similar mechanism also was proposed to regulate H4 acetylation, transcription of the gene encoding the BK-type potassium channel, and tolerance to benzyl alcohol in the fruit fly, Drosophila (Wang et al. 2007).

Gene expression experiments have provided additional support for the role of histone acetylation in alcohol addiction. Several studies focusing on brain changes in human alcoholics have shown general downregulation of genes involved in histone acetylation and upregulation of genes promoting histone deacetylation. The latter group of genes includes those encoding proteins forming so-called transcription corepressor complexes (TCCs), which help suppress transcription by coupling HDAC activity with DNA methylation, thereby establishing a repressive chromatin state (McDonel et al. 2009). For example, transcripts of the genes encoding CREB and CBP were down-regulated in alcoholics (Ponomarev et al. 2012). Conversely, transcripts of the gene *MBD3*, which encodes a key player in TCCs called methyl-CpG–binding protein, as well as many other TCC genes, such as *SIN3A, SIN3B*, player in TCCs called methyl-CpG– binding protein, as well as many other TCC genes, such as *SIN3A, SIN3B, MTA1, MTA2, RBBP4, GATAD2A, GATAD2B*, and *CHD4* were upregulated in alcoholics (Liu et al. 2006; Ponomarev et al. 2012; Zhou et al. 2011). Together, these observations validate previous findings that histone acetylation is decreased during alcohol withdrawal (Pandey et al. 2008) and suggest that TCCs are activated and play a role in the downregulation of some genes in the alcoholic brain.

## MicroRNAs

MicroRNAs (miRNAs) comprise a specific class of noncoding RNAs that bind to complementary sequences on target mRNAs to repress translation and silence gene expression (Robison and Nestler 2011). Expression of miRNAs can alter the transcriptional potential of a gene in the absence of any change to the DNA sequence and therefore can be considered an epigenetic phenomenon. The most convincing evidence for the involvement of miRNAs in alcohol-related gene expression was presented by Pietrzykowski and colleagues (2008), who showed that alcohol upregulates expression of microRNA 9 (miR-9) in rat brain, which results in miR-9–dependent downregulation of BK channel variants with high sensitivity to alcohol. This mechanism is proposed to mediate the development of cellular tolerance and generally may contribute to neuronal adaptation to alcohol.

Additional evidence for the role of miRNAs in alcohol-induced regulation of gene expression and behavior comes from genomic studies measuring levels of multiple miRNAs after exposure to alcohol. Using neural cultures and a model of alcohol-induced teratogenesis, Sathyan and colleagues (2007) identified the first alcohol-sensitive miRNAs. Subsequent studies using miRNA microarrays detected multiple alcohol-regulated miRNAs in neural cultures (Yadav et al. 2011), fetal mouse brains (Wang et al. 2009), and brains of human alcoholics (Lewohl et al. 2011).

## Summary and Future Directions

The findings reviewed in this article point to a central role of various epigenetic processes in controlling alcohol-induced changes in brain gene expression and behavior, which may play an important part in the development of alcohol addiction (see the figure). For example, chronic alcohol exposure can result in global DNA hypomethylation via several mechanisms, including vita-min B and folate deficiencies that can lead to an impairment of one-carbon metabolism and a decrease in SAM levels. However, these global effects of alcohol do not imply unidirectional changes across the whole genome, because many genes show the opposite epigenetic changes in their promoters.

Many of the observed chromatin modifications are mechanistically linked, resulting in a limited number of chromatin states (Jaenisch and Bird 2003). For example, trimethylation of H3K4 is mechanistically coupled with unmethylated DNA (Hashimoto et al. 2010), suggesting that the reduced DNA methylation observed in alcoholic brains can promote a general increase in the H3K4me3 levels. Histone actetylation patterns also are commonly altered by alcohol in a process that may be linked to DNA methylation. Thus, acute alcohol exposure promotes his-tone acetylation, whereas withdrawal from chronic alcohol often increases deacetylation of histones. Deacetylation via HDAC activity is coupled to DNA methylation through the actions of methyl-binding proteins and other TCC components. Chronic alcohol exposure leads to upregulation of TCC genes, which may serve to compensate for the reduced number of methylated CpGs. These cumulative changes in ferent cell types and lead to activation of microglia, neuronal degeneration, and compensatory neuroadaptations in alcoholic brain. In summary, alcohol-induced epigenetically mediated changes in gene expression may underlie the brain pathology and adaptations in brain functioning (i.e., brain plasticity) associated with alcohol abuse and alcohol dependence and may contribute to alcohol relapse and craving.

To advance the current state of epigenetic research in alcoholism, future studies that look at both simplified models and entire regulatory systems (i.e., that use both reductionist and systems approaches) are needed. One focus of this research should be on understanding the exact mechanistic links between chronic alcohol exposure, epigenetic changes, and gene expression. Exploratory studies likely will first use discovery-driven approaches to investigate the mechanistic relationships between the epigenome and the transcriptome in animal models and formulate hypotheses at the single-gene, gene-network, and systems levels. Follow-up studies using both animal models and human postmortem material then can help test these hypotheses and validate functional predictions of the genome-wide experiments. Because of the complex temporal relationships between chromatin marks and transcriptional changes, time-course studies also will be required. The recently available epigenetic maps from the ENCODE (ENCyclopedia Of DNA Elements) Project (Dunham et al. 2012) should help accelerate these research efforts.

To address the causal relationships between epigenetic modifications and alcohol traits, it will be essential to use tools of both forward and reverse genetics. Forward-genetics approaches seek to determine the genetic basis of an observed trait (i.e., phenotype). Such approaches include mapping DNA regions that may contain disease-related genes (i.e., quantitative trait loci [QTLs]), using chromatin modifications as phenotypes. This can be achieved using genetic reference panels, such as recombinant inbred strains of mice and rats (Rosen et al. 2007). Many reference populations have been tested extensively for both expression of specific genes and alcohol-related behaviors and therefore can serve as powerful tools for integrating data across biological modalities and investigating mechanistic links between the genome and the entirety of all analyzed phenotypes (i.e., the phenome) through genetic mapping of the epigenome and the transcriptome. Conversely, reverse-genetics approaches study the phenotypes that arise as the result of alterations of particular genes. An example of a reverse-genetics approach is to assess alcohol-related behaviors in mice with genetic mutations of chromatin-binding proteins.

Another important research direction is to investigate the cellular specificity of alcohol-induced epigenetic changes. For example, future research should determine cell type–specific chromatin states that drive the unique molecular responses to alcohol in different neurons and glial cells and show how epigenetic modifications help establish functional states consistent with the pathophysiological changes observed in alcoholism. One example of this approach is the analysis of the role of epigenetically controlled ERVs in alcohol addiction (Ponomarev et al. 2012). Previous studies found that an ERV-encoded glycoprotein called syncytin can directly activate different types of glial cells (i.e., microglia and astrocytes) and induce neuroinflammation (Antony et al. 2004). Microglial activation, in turn, can result in neuronal degeneration (Crews et al. 2011), and syncytin-activated astrocytes can secrete compounds that are toxic to other glial cells (i.e., oligodendrocytes) and thus lead to myelin degeneration (Antony et al. 2004). Both of these effects are consistent with pathologies observed in alcoholics (Harper et al. 2003; Pfefferbaum et al. 2009; Zahr et al. 2011). Alcohol-induced neuroimmune responses have been suggested to be a critical factor in alcohol addiction (Crews et al. 2011), and Ponomarev and colleagues (2012) proposed a novel mechanism including the potential role for ERVs in neuroinflammation and brain pathophysiology of human alcoholism. Another approach to assessing the cell specificity of epigenetic processes is to compare alcohol-induced epigenetic changes across tissues and cell types. Human research often is limited to peripheral tissues (e.g., blood). To be able to draw parallels between peripheral and central mechanisms in humans, researchers first need to study the relationships between responses to alcohol in the brain and those in other tissues using animal models.

Other research efforts should focus on the potential exploitation of epigenetic mechanisms for alcoholism treatment. Epigenetic therapeutics, such as HDACis, offer unique advantages in treating diseases through chromatin-dependent changes in gene expression. These “master regulators” can affect expression of multiple genes. Therefore, in order to understand the effects of these agents on alcohol behaviors, it is important to study their mechanisms of action and identify the range of genes and molecular pathways affected. Large-scale genomic studies should focus on the global relationships between chromatin marks and gene expression in the context of chronic alcohol exposure and epigenetic therapeutics. Finally, multiple studies in humans and animal models have highlighted the importance of the genetic component in alcohol addiction (Crabbe 2008; Mayfield et al. 2008; Spanagel 2009). To better understand the interplay between genetic, epigenetic, and environmental factors in controlling gene expression in alcoholism, integrative approaches across studies are warranted. Many epigenetic therapeutics have been developed for other diseases, and understanding the functional relationships between epigenetic processes and the transcriptome in the alcoholic brain may lead to new molecular targets for medication development for human alcoholism.

## Figures and Tables

**Figure:**
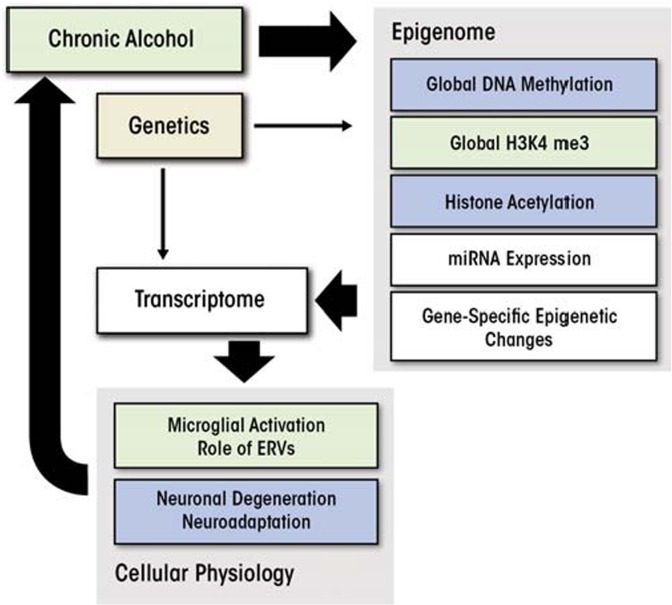
A hypothetical diagram for the role of epigenetic modifications in alcohol addiction. Yellow color indicates general increase, up-regulation, or activation, whereas blue color indicates general decrease, down-regulation, or degeneration. White background implies bidirectional changes. Potential interactions between different components of the diagram are discussed in the text.

## Footnotes

1 The first step of gene expression (i.e., transcription) involves the synthesis of intermediary molecules called messenger RNAs (mRNAs) that are copies of the gene(s) to be expressed and which serve as templates for the synthesis of the encoded protein(s) during the second step of gene expression (i.e., translation). A transcriptome is the entirety of all mRNAs found in a certain cell, organ, or organism.

2 The dose administered was 6 g/kg of a 32 percent ethanol solution.
